# Evidence-based commentary on the diagnosis, management, and further research of degenerative cervical spinal cord compression in the absence of clinical symptoms of myelopathy

**DOI:** 10.3389/fneur.2024.1341371

**Published:** 2024-05-10

**Authors:** Tomas Horak, Magda Horakova, Milos Kerkovsky, Marek Dostal, Petr Hlustik, Jan Valosek, Alena Svatkova, Petr Bednarik, Eva Vlckova, Josef Bednarik

**Affiliations:** ^1^Faculty of Medicine, Masaryk University, Brno, Czechia; ^2^Department of Neurology, University Hospital Brno, Brno, Czechia; ^3^Department of Radiology and Nuclear Medicine, University Hospital Brno, Brno, Czechia; ^4^Department of Biophysics, Faculty of Medicine, Masaryk University, Brno, Czechia; ^5^Department of Neurology, Faculty of Medicine and Dentistry, Palacký University Olomouc, Olomouc, Czechia; ^6^Department of Neurology, University Hospital Olomouc, Olomouc, Czechia; ^7^Department of Neurosurgery, Faculty of Medicine and Dentistry, Palacký University Olomouc, Olomouc, Czechia; ^8^NeuroPoly Lab, Institute of Biomedical Engineering, Polytechnique Montreal, Montreal, QC, Canada; ^9^Mila—Quebec AI Institute, Montreal, QC, Canada; ^10^Danish Research Center for Magnetic Resonance, Center for Functional and Diagnostic Imaging and Research, Copenhagen University Hospital Amager and Hvidovre, Copenhagen, Denmark; ^11^Department of Radiology, Center for Functional and Diagnostic Imaging and Research, Copenhagen University Hospital Amager and Hvidovre, Copenhagen, Denmark

**Keywords:** degenerative cervical cord compression, degenerative cervical myelopathy, cervical spinal canal stenosis, magnetic resonance imaging, subclinical myelopathy

## Abstract

Degenerative cervical myelopathy (DCM) represents the final consequence of a series of degenerative changes in the cervical spine, resulting in cervical spinal canal stenosis and mechanical stress on the cervical spinal cord. This process leads to subsequent pathophysiological processes in the spinal cord tissues. The primary mechanism of injury is degenerative compression of the cervical spinal cord, detectable by magnetic resonance imaging (MRI), serving as a hallmark for diagnosing DCM. However, the relative resilience of the cervical spinal cord to mechanical compression leads to clinical-radiological discordance, i.e., some individuals may exhibit MRI findings of DCC without the clinical signs and symptoms of myelopathy. This degenerative compression of the cervical spinal cord without clinical signs of myelopathy, potentially serving as a precursor to the development of DCM, remains a somewhat controversial topic. In this review article, we elaborate on and provide commentary on the terminology, epidemiology, natural course, diagnosis, predictive value, risks, and practical management of this condition—all of which are subjects of ongoing debate.

## 1 Introduction

**Figure 1:**
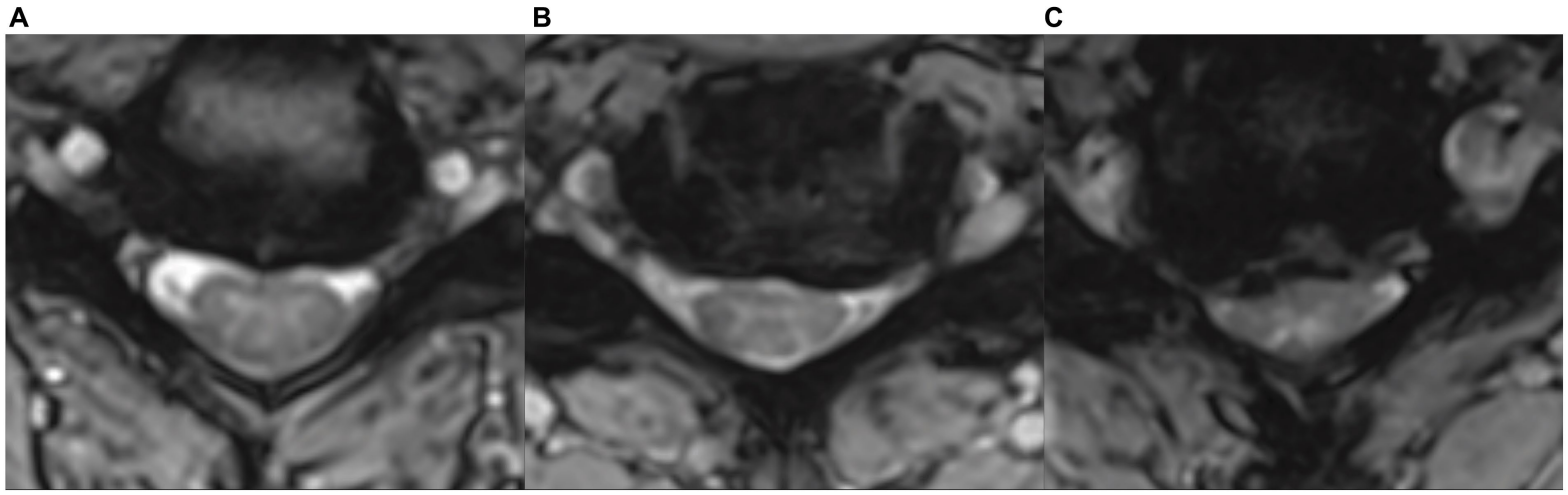
Examples of different types and severities of cervical spinal cord compression in axial MRI images in asymptomatic degenerative cervical cord compression subjects, illustrating the weak correlation between the severity of compression and the development of clinical myelopathic symptoms and signs. **(A)** Small ventral focal compression („impingement“) with preserved cerebrospinal fluid space. **(B)** Flat ventral compression with flattened spinal cord and partially reduced ventral cerebrospinal fluid space. **(C)** Severe circular asymmetrical compression with flattening of the spinal cord and almost complete loss of cerebrospinal fluid space.

**Figure 2:**
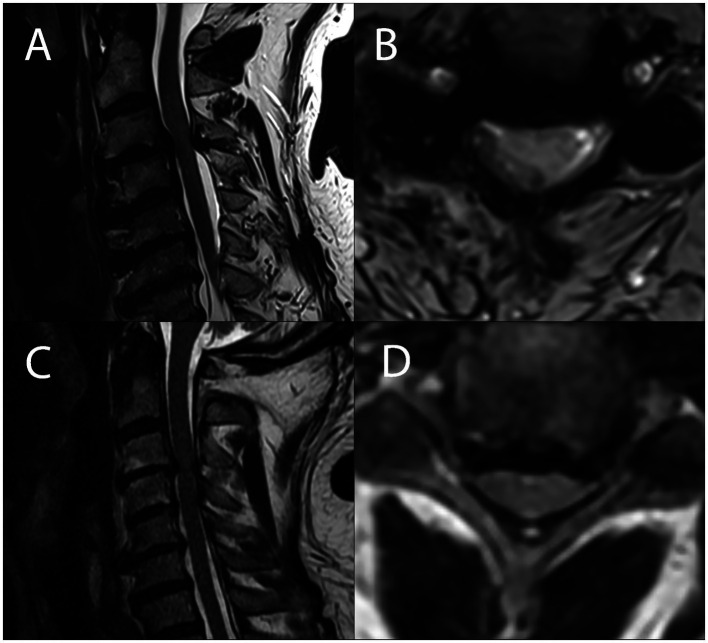
Sagittal and axial MRI sections of the cervical spinal cord showing degenerative compression in a patient with symptomatic degenerative cervical myelopathy **(A,B)** and in a patient without any myelopathic signs and symptoms **(C,D)**, showing no visible difference in the severity of compression between DCM and ADCC subjects.

Degenerative changes in the cervical spine, primarily involving spondylosis and discopathy, lead to the narrowing of the cervical spinal canal, known as cervical spinal canal stenosis (CS). These changes are considered a common aspect of the aging process and are prevalent among the elderly population (1). The most serious and disabling consequence of CS is degenerative cervical myelopathy (DCM). DCM is an umbrella term used to describe progressive compression of the cervical spinal cord due to age-related changes in the spinal axis (2). Mechanical degenerative cervical spinal cord compression (DCC) is a key mechanism in the development of DCM, which together with complex pathophysiological mechanisms (e.g., necrosis, inflammation, gliosis, edema, demyelination, ischemia, and axonal and neuronal loss) leads to a variety of myelopathic symptoms. Current pathobiological and mechanistic knowledge does not adequately explain the disease phenotype, why only a subset of patients with identified spinal cord compression have clinical myelopathy, or why the degree of spinal cord compression correlates poorly with clinical disability. It has been proposed that DCM is better described as a function of multiple interacting mechanical forces, such as shear, traction, and compression, together with an individual’s susceptibility to spinal cord injury, influenced by factors such as age, genetics, cardiovascular, gastrointestinal, and nervous system status, and time (3). Unlike compression, which can be visualized by MRI, the other mechanical forces are difficult to document or quantify. MRI evidence of spinal cord compression is therefore a key element in the diagnosis of DCM, along with clinical signs and symptoms of myelopathy (4, 5). Using the presence of clinical signs and symptoms of myelopathy as the main diagnostic criterion for the diagnosis of DCM can be difficult and sometimes misleading, as clinical signs of myelopathy can be present in a wide range of other diseases (2). Furthermore, the relative resilience of the cervical spinal cord to mechanical compression leads to a relatively high prevalence of clinical-radiological discordance, i.e., some individuals may exhibit MRI findings of DCC without the clinical signs and symptoms of myelopathy (Figures 1, 2). This condition may eventually progress to symptomatic DCM and should be considered as a precursor to DCM.

Several aspects of this condition need clarification and should be addressed in further research. First, there needs to be a general agreement on its definition and terminology, possibly encouraging further research into the subject. Second, the prevalence and natural history should be known. Third, as this probably common condition may precede the development of the much rarer DCM, it would be appropriate to identify biomarkers of the higher risk of progression to promote optimal management of high-risk individuals. Finally, the risk of developing symptomatic myelopathy after minor trauma, a risk that may eventually lead to a recommendation for surgery, should be disclosed.

## 2 Definition and terminology

Proper clinical management and even research into DCC without symptomatic myelopathy is hampered by its inconsistent definition and terminology, partly due to overlap with CS on the one hand and DCM on the other.

Cervical spinal stenosis is undoubtedly a key element leading to the eventual development of symptomatic radiculopathy or myelopathy. However, it is primarily defined as an anatomical narrowing of the cervical spinal canal that may lead to DCM but can and usually does remain completely asymptomatic for a long time or throughout life. Several measures are used to define both “developmental” and “degenerative” CS. Anteroposterior canal diameter < 10 mm or a Torg-Pavlov ratio < 0.82, as measures of narrowing of the bony cervical canal, tend to reflect congenital stenosis (6–8). Several more sophisticated measures have been proposed to define and quantify degenerative CS, such as “osseous spinal canal area,” “dural sac area” (7), “space available for cord,” and “canal to cord ratio” (9). However, the degree of CS correlates only weakly with the development of DCM, as we discuss later. CS is often presented as interchangeable with DCC (10, 11), which can be somewhat misleading. It seems logical to keep the term CS as an anatomical signature of the narrowing of the cervical spinal canal. Subjects with CS may have cervical spinal cord compression and clinical signs and symptoms of clinically symptomatic DCM, radiculopathy, cervical pain, and limited range of neck motion, or they may remain completely asymptomatic.

A recent review of the literature found 118 articles on the pathology preceding DCM (12). The most common term found was asymptomatic (88%), followed by non-myelopathic (26%), presymptomatic (11%), subclinical (5%), and silent (2%). The greatest inconsistency was in the use and definition of “asymptomatic,” with some papers using the term synonymously with healthy controls; the majority used it to describe patients with radiological evidence of degenerative spinal compression or other pathology, but without clinical symptoms of myelopathy. There was a further discrepancy between patients with and without symptoms and/or signs of radiculopathy (12).

The key question remains which conditions precede DCM and should be distinguished, defined, and appropriately named. It seems useful not to confuse simple CS with cases of radiologically proven DCC. Both of the most commonly used terms have some disadvantages. The term “asymptomatic” refers to the absence of clinical signs or symptoms of clinically symptomatic myelopathy, but some cases may have symptoms of radiculopathy or cervical pain and are therefore not completely asymptomatic. The term “non-myelopathic” may resolve this discrepancy, but it may also lead to the false assumption that there is no spinal cord injury.

**Figure 3:**
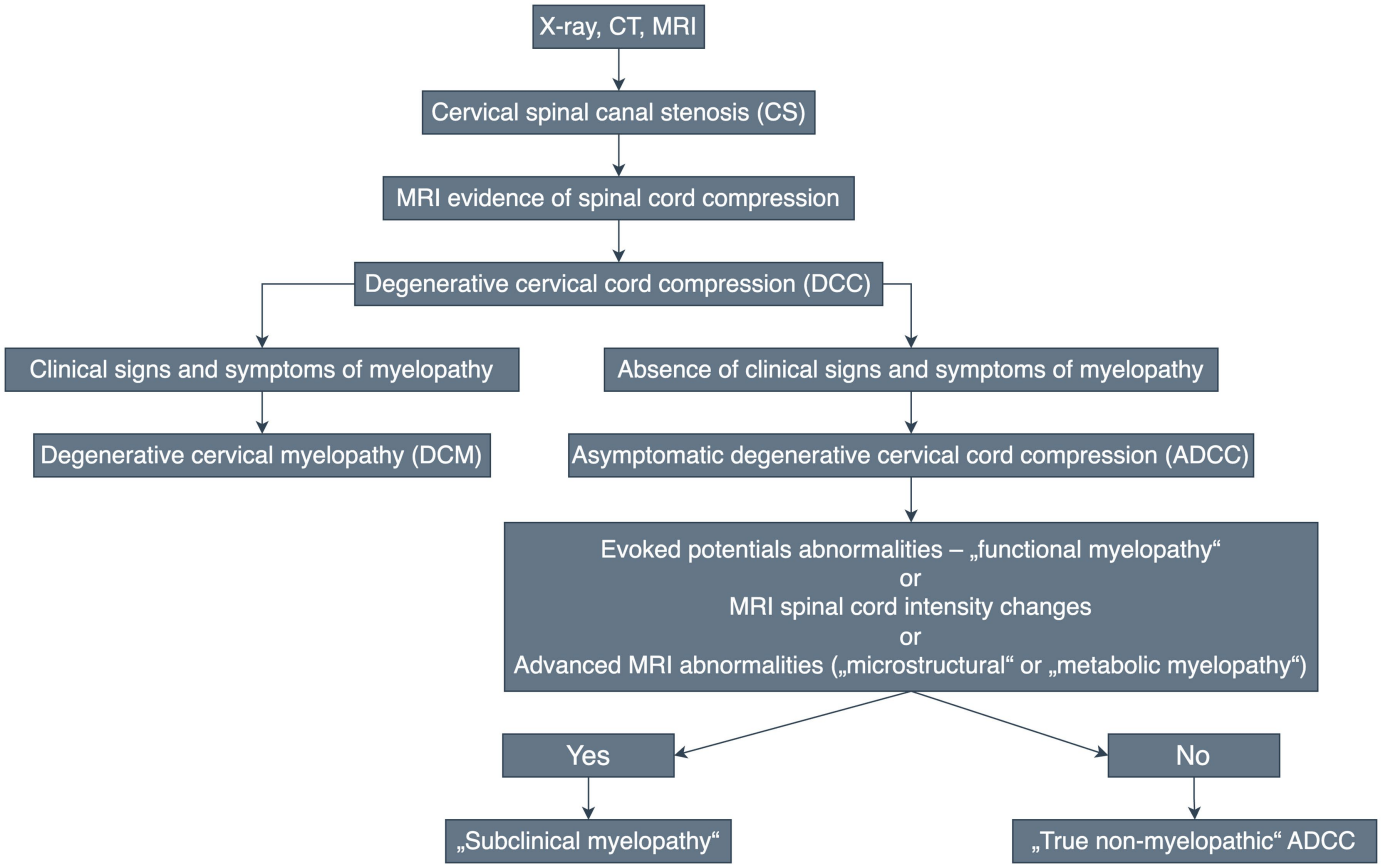
Diagram of the diagnosis and terminology of degenerative cervical spinal canal stenosis and subsequent cervical spinal cord compression.

It seems useful to coin the term asymptomatic degenerative cervical cord compression (ADCC) to describe individuals with radiological evidence of degenerative compression of the spinal cord, and to further stratify this group with respect to the presence or absence of symptomatic radiculopathy (13) or the presence of spinal cord dysfunction (detected by electrophysiological methods) or microstructural or metabolic myelopathy (detected by advanced MRI techniques, as we discuss later)—Figure 3.

### 2.1 Recommendation

The term “asymptomatic degenerative cervical cord compression” (ADCC) should preferably be used to describe individuals with MRI evidence of degenerative cervical cord compression without clinical signs and symptoms of myelopathy. The term “non-myelopathic degenerative cervical cord compression” should be considered synonymous with ADCC. The term cervical spinal stenosis (CS) should be reserved for describing anatomical narrowing of the cervical spinal canal, both developmental and degenerative.

## 3 Epidemiology and natural history

Degenerative cervical myelopathy, despite its low prevalence, cannot be categorized as a rare disease, as it is the most common cause of non-traumatic cervical spinal cord injury and lower limb paraparesis in individuals aged 55 and above (14). In North America, the published annual incidence is 41 per 1 million, and the prevalence is 605 per 1 million (15, 16). Nonetheless, a systematic review estimated the prevalence of DCM in the population to be as high as 2.3% (17).

The estimated prevalence of ADCC in a healthy population is much higher. A meta-analysis and systematic review demonstrated an estimated prevalence of 24.2%, with a significantly higher prevalence of ADCC in older populations and in North American/European populations as compared to Asian populations. In White European/North American populations aged over 60, the prevalence has risen as high as 40% (17, 18). A recent study of 267 young adult volunteers (with a mean age of 28.7 ± 5.6) identified the presence of mild spinal cord compression in 24% of the participants (19). ADCC is therefore a very common condition and a possible precursor of DCM, which increases the importance of its clinical management.

The natural history of ADCC is a key factor in assessing the risk of developing DCM and the influence of potential factors that increase this risk. In a small imaging study of 20 ADCC individuals, 2 (10%) eventually developed symptoms of myelopathy at a median follow-up of 21 months (20). In the largest prospective study performed to date on this topic, of 199 patients enrolled with ADCC, 8% developed symptoms of myelopathy at 12 months and 22.6% developed symptoms of myelopathy at a median follow-up of 44 months (range 12–24 months) (21). In another study by the same group in 2017, 13.4% of patients (15/112) developed DCM at a median follow-up of 36 months (22).

### 3.1 Recommendation

Asymptomatic degenerative cervical cord compression should be considered a very common condition, especially in an elderly White population. The rate of progression to DCM in the short and medium term is likely to be relatively low, not exceeding a few percent per year.

## 4 Detecting degenerative cervical cord compression

Magnetic resonance imaging is the reference imaging modality for assessing the extent of spinal cord compromise or injury, and typical features include visible spinal cord compression, altered spinal cord signal intensity, CS, altered sagittal spinal alignment, and ligamentous changes. Various quantitative measures of spinal cord compromise have been described, including “transverse area,” “compression ratio,” “maximum spinal cord compression,” and “spinal cord occupation ratio.” Despite years of research, no standard MRI features have been found to consistently represent disease severity in DCM (23). Even the extent of degenerative cervical cord compression, considered a hallmark of cervical cord injury in DCM, correlates poorly with the severity of clinical involvement (23, 24) (Figure 1). Nevertheless, the detection of DCC is critical to the current correct definition and diagnosis of both DCM and ADCC, and non-specialists, in particular, need information based on a reliable and consistent definition of MRI evidence of DCC, optimally provided by routine MRI.

The imaging definitions of DCC based on both quantitative and qualitative methods are vague, with no generally accepted quantitative parameter as a hallmark of DCC. The criteria used for DCC vary between studies, leading to bias in meta-analyses and making multicenter studies difficult (17, 25). Additionally, repeated MRI in longitudinal follow-up of mild DCM and ADCC requires reliable quantitative measures to assess the potential progression of radiological outcomes. Manual assessment of these quantitative measures is time-consuming and prone to inter-rater variability, making it unsuitable for large longitudinal studies. In 2014, the Spinal Cord Toolbox (SCT), an open-source software package for the analysis of spinal cord MRI data was introduced (26). Among its many functionalities, the SCT offers automatic spinal cord segmentation and morphometric analysis tools (26, 27) enabling automatic extraction of common radiological measures such as transverse diameter, anterior–posterior diameter, and cross-sectional area (CSA), as well as parameters reflecting cord indentation and torsion. Martin et al. (20) recently compared the automatic shape analysis of morphometric measures calculated by SCT with expert assessments and reported promising results. They also proposed an objective definition of DCC as a deviation from normal in one of three quantitative parameters reflecting flattening, indentation, and torsion. Morphometric measures semi-automatically derived from two MRI scans using the SCT demonstrated the ability to detect spinal cord compression based on four parameters (CSA, solidity, compression ratio, and torsion) with lower inter-trial variability than a manual assessment by three experts (25). Despite the promising results, additional studies are needed to verify the generalization of the proposed methodologies across different MRI scanners, sequence settings, and population cohorts. The recently released *spine-generic* MRI acquisition protocol (28, 29) and morphometric measure normalization (19) can be employed to standardize both the MRI data acquisition and morphometry analysis in future multicenter and longitudinal studies.

### 4.1 Recommendation

The terminology used to describe spinal cord compression in radiological reports should be standardized. An automatic quantitative method of detecting spinal cord compression based on routine MRI sequences and freely available software may be helpful and potentially facilitate the practical management of both ADCC and DCM.

## 5 “Subclinical myelopathy” identified by imaging techniques in patients with DCC

A key diagnostic criterion for DCM (in addition to MRI evidence of cervical cord compression) is the presence of clinical symptoms and signs of myelopathy (4, 5). The use of this criterion can be difficult and sometimes misleading, as symptoms of DCM can also be present in a wide range of other conditions (2). However, the development of clinical myelopathic symptoms or signs can be a rather insensitive and late marker of spinal cord injury. Moreover, in quite a significant proportion of individuals with DCC but without clinical signs and symptoms of myelopathy, it is possible to detect subclinical functional, metabolic, and microstructural abnormalities using advanced or even routine diagnostic methods.

Electrophysiological methods, in particular, somatosensory evoked potentials (SEPs) and motor evoked potentials (MEPs) and electromyography can detect functional abnormalities of the spinal cord pathways or anterior horn cells in ADCC subjects (21, 30). Contact heat evoked potentials (CHEPs) have been shown to be more sensitive in detecting sensory pathway abnormalities in DCM (31) but have not been systematically studied in individuals with ADCC.

In addition to routine MRI showing “macrostructural” T2 or T1 hyper/hypointensities in the spinal cord, several novel quantitative MRI techniques are able to detect evidence of microstructural or metabolic myelopathy in patients with ADCC (32) using diffusion MRI (dMRI) (20, 33–37), T2*-weighted white/gray matter signal intensity ratio (38, 39), voxel-based volumetry demonstrating spinal cord degeneration (40–42), or proton (1H) magnetic resonance spectroscopy (MRS) (43) in comparison with healthy controls. While dMRI in ADCC patients consistently detected lower fractional anisotropy and higher mean diffusivity at compressed levels, caused by demyelination and axonal injury (20, 33, 34), magnetization transfer and 1H-MRS, along with advanced and tract-specific dMRI, recently revealed microstructural alterations, also rostrally pointing to Wallerian degeneration (19, 20, 36, 43). Recent studies also disclosed a significant relationship between microstructural damage and functional deficits, as assessed by quantitative MRI and electrophysiology, respectively (36, 43). Thus, tract-specific quantitative MRI, in combination with electrophysiology, critically extends our understanding of the underlying pathophysiology of degenerative spinal cord compression and may provide predictive markers of DCM development for accurate patient management. However, the prognostic value must be validated in longitudinal studies. The increased availability of 3 T MRI machines has facilitated the practical use of these techniques.

### 5.1 Recommendation

It seems reasonable to refer to ADCC patients with the evidence of “microstructural,” “metabolic,” or “functional” cervical cord impairment as ADCC with “subclinical myelopathy,” whereas those with no other MRI abnormality (other than spinal cord compression) and no evidence of electrophysiological spinal cord dysfunction can be referred to as “true non-myelopathic” ADCC subjects (Figure 1). The predictive value of the presence of subclinical myelopathy detected by imaging techniques in ADCC subjects for progression to DCM should be established.

## 6 Which ADCC individuals have a higher risk of developing DCM?

The presence of radiculopathy and dysfunction of spinal cord pathways detected by evoked potentials have been repeatedly reported to predict a higher risk of progression to symptomatic DCM (21, 22, 30), but practical recommendations for the management of ADCC subjects are still debated (11, 13).

As for the imaging (mostly MRI) predictors of progression to the symptomatic myelopathy stage, many of these parameters have been studied, but with inconsistent results (24).

Older criteria for defining a narrow spinal canal, also known as “congenital canal stenosis” or “developmental canal stenosis,” based on radiographic and cadaveric studies, used a sagittal width of <12–13 mm or a Torg-Pavlov ratio of <0.80–0.82 for diagnosis (44, 45), but evidence supporting a clear association between congenital stenosis and the development of myelopathy remains sparse (46). Recently, the relative size of the canal and spinal cord has been assessed, with the assumption that both a narrow canal and a large spinal cord may predispose patients to cervical spinal cord compression and potential myelopathy development (47, 48). This knowledge has led to the development of relative parameters based on MRI data that incorporate the size of the spinal cord, including space available for the cord (SAC) and spinal cord occupation ratio (SCOR). Depending on the technique, a cord-canal mismatch can be defined as a SCOR ≥70% when measured on the midsagittal plane (49), ≥80% in the axial plane (50), or < 5 mm of SAC (51). In the subanalysis of the international and multicenter AOSpine studies of surgically treated patients with DCM, the prevalence of a cord-canal mismatch using a sagittal SCOR ≥70% was 8.4%, and patients diagnosed with a cord-canal mismatch at non-compressed sites were 5.4 years younger and had reduced baseline neurological function and quality of life (49). While both the large cord and the smaller canal have been shown to be risk factors for DCM, the predictive value of these parameters describing cord-canal mismatch and measured outside the level of compression for the development of DCM has not been investigated in the ADCC population.

Measures of the severity of cervical cord compression, such as compression ratio and CSA, have primarily been used to define and detect cord compression itself but have only exceptionally been studied as predictors of the development of DCM in ADCC individuals (22). In a 36-month longitudinal follow-up study of 112 ADCC individuals, multivariate analysis showed that radiculopathy, axial CSA ≤ 70.1 mm^2^, and compression ratio ≤ 0.4 were predictive of the development of DCM.

Intramedullary signal changes in the spinal cord are commonly observed in patients with DCC, and the prevalence of T2 hyperintensity has been reported to range from 58 to 85% in patients with clinical symptoms of myelopathy (52). There appears to be a graded increase in neurological impairment when comparing patients with no signal changes, T2 hyperintensity, and both T2 hyperintensity and T1 hypointensity (53, 54). However, similar hyperintensity may also be an incidental finding. It was observed that 2.3% of 1,211 asymptomatic subjects had evidence of compressive cervical spine pathology with associated T2 hyperintensity (47). A few studies have investigated the predictive value of intramedullary signal changes on MR imaging in patients with mild DCM treated conservatively. Shimomura et al. found that T2 hyperintensity was not predictive of clinical progression as measured by worsening Japanese Orthopedic Association (JOA) score in patients with mild myelopathy (Shimomura). The predictive value of T2 hyperintensity in ADCC subjects has not been systematically studied. According to a review by Wilson et al. (11), hyperintensity on a T2-weighted MRI is a significant predictor of myelopathy development.

Several novel quantitative MRI techniques are able to detect evidence of microstructural or metabolic myelopathy in ADCC subjects, as discussed above, but their predictive value for progression to DCM has not been systematically investigated.

There are potential or established risk factors (including genetic and environmental) for the development of DCM, which have recently been summarized (3, 24). However, these are general risk factors for the development of DCM, but it is not known whether these risk factors, if assessed, could predict further outcome scenarios in pre-existing asymptomatic degenerative cervical cord compression.

### 6.1 Recommendation

It is reasonable to evaluate the contribution of both established clinical and electrophysiological predictors (i.e., radiculopathy and electrophysiological abnormalities) together with the new promising potential imaging predictors reflecting the severity of compression or subclinical microstructural or metabolic myelopathy in future longitudinal studies.

We propose that ADCC patients with identified high-level risk factors for developing DCM (including radiculopathy) be referred to as “presymptomatic myelopathy” subjects.

## 7 Risk of traumatic injury in ADCC

It is not uncommon for patients with ADCC, or even those with radiographic cervical spinal stenosis, to be recommended for surgery to reduce a perceived increased risk of neurological injury from a traumatic event (55). This problem is even more pressing in athletes or people accustomed to high-risk activities. However, the current literature addressing this issue is controversial.

A prospective cohort of 199 patients with ADCC was reviewed to specifically assess whether trauma is a risk factor for the development of neurological impairment (56). Fourteen traumatic events were identified during a mean follow-up period of 44 months, and only three minor traumatic events without cervical spine fracture were found among the symptomatic myelopathy cases, with no chronological relationship between trauma and myelopathy. The authors concluded that the risk of spinal cord injury is likely to be low, especially if a restriction on high-risk activities is implemented. This finding was supported by Chang et al. (57), who found that in a cohort of 55 prospectively followed asymptomatic or mildly symptomatic patients with CS, 18% experienced a traumatic event, but none of these had evidence of a spinal cord injury.

In another study, Ruegg et al. (50) used a retrospective case control methodology to address this question. A consecutive cohort of 52 patients presenting to a single center with traumatic quadriplegia or quadriparesis following a minor event over a 10-year period was compared with controls with similar minor injuries but no associated neurological compromise. They found that patients at risk of acute spinal cord injury after mild trauma can be reliably identified using the cord-canal-area ratio (>0.8) or the space available for the cord (<1.2 mm) measured on MRI. However, caution should be exercised before extrapolating these findings to all people with asymptomatic ADCC. No details are given, but the authors suggest that they excluded people with preexisting neurological symptoms. It is possible that some patients with post-traumatic neurological injury may have had preexisting symptoms of myelopathy that were not identified, given the retrospective nature of this study. It is noteworthy that falls were the precipitating mechanism in almost twice as many cases with neurological injury as in the controls (48% of cases vs. 27% of controls), which may indicate preexisting, yet unrecognized symptoms of myelopathy (55).

In a recent review article, a Torg-Pavlov ratio < 0.7, a minimal disk-level canal diameter < 8 mm, a cord-to-canal area ratio > 0.8, or space available for the cord <1.2 mm, were suggested as markers of higher risk for cervical spinal injury due to a traumatic event in patients with “asymptomatic cervical canal stenosis.” These criteria were thought to be particularly useful in advising people who play either contact or collision sports (10).

### 7.1 Recommendation

Counseling ADCC subjects to avoid high-risk activities should be considered.

A high-quality prospective controlled study should be conducted to clarify the potential increased risk of spinal cord injury after minor injury in the ADCC population. If such a risk is documented, the subsequent benefit of surgery to reduce this risk should be demonstrated.

## 8 Practical management of ADCC

In the first comprehensive systematic review and survey on patients with ADCC (11), a series of recommendations were made regarding the frequency, timing, and predictors of myelopathy development in asymptomatic patients with ADCC based on five articles that met most of the inclusion criteria of the review. They suggested that patients with ADCC who have clinical or electrophysiological evidence of cervical radicular dysfunction or central conduction deficits appear to be at higher risk for developing myelopathy and should be counseled to consider surgical treatment.

In subsequent AO Spine guidelines (13), this recommendation was further elaborated and modified. Patients with ADCC with clinical evidence of radiculopathy, with or without electrophysiological confirmation, are considered to be at a higher risk of developing myelopathy and should be counseled about this risk. These patients should be offered either surgical intervention or non-operative management consisting of close serial follow-up or a supervised trial of structured rehabilitation.

An evidence-based commentary (55) confirmed the lack of evidence to support surgery in asymptomatic individuals with ADCC who have no risk factors for progression. For these patients, the authors suggest nonoperative management, including education on the symptoms of myelopathy, clinical follow-up within 6–12 months, and avoidance of high-risk activities.

There is no clear recommendation on the extent of follow up, including the use of electrophysiological assessment or advanced MRI techniques to detect subclinical myelopathy, or repeated MRI to document the progression of DCC.

### 8.1 Recommendation

Patients with ADCC should be educated about the symptoms associated with myelopathy and should have follow-up visits on a regular basis, at least at 1-year intervals.

Patients with ADCC at higher risk of developing DCM (those currently with clinically symptomatic radiculopathy) should be offered surgery with counseling on both the risk of progression and the risk of surgery. The higher risk of developing DCM in “subclinical myelopathy” needs to be confirmed.

The optimal practical management of patients with ADCC, including the frequency, duration, and extent of clinical surveillance, additional testing, and avoidance of high-risk activities, should be further discussed and reviewed.

## 9 Conclusion

Asymptomatic degenerative cervical cord compression is a very common condition, particularly in the elderly population. A proportion of ADCC subjects may have some clinical non-myelopathic symptoms and signs of radiculopathy or cervical pain, or may have evidence of subclinical “microstructural,” “metabolic,” or “functional” cervical cord impairment detected by electrophysiological or advanced MRI techniques, while a large proportion may remain free of any clinical signs and symptoms, any MRI abnormality (other than spinal cord compression) and any evidence of electrophysiological spinal cord dysfunction (“true non-myelopathic” ADCC subjects). Further research is necessary to enhance the understanding of the natural history and the rates of deterioration of ADCC. This includes identifying important biomarkers (such as clinical, imaging, and electrophysiological factors) that predict clinical outcomes, improving clinical communication, facilitating treatment decisions, and determining the optimal duration and frequency of follow-up. The advancement of standardized classification and terminology, the standardization of MRI analysis and processing, and a critical analysis of the somewhat controversial existing evidence, are crucial for guiding both research and clinical recommendations in the significant yet sometimes overlooked area of ADCC, which acts as a precursor to DCM.

## Author contributions

TH: Project administration, Writing – original draft. MH: Writing – review & editing. MK: Writing – review & editing, Methodology. MD: Writing – review & editing. PH: Supervision, Writing – review & editing. JV: Methodology, Writing – review & editing. AS: Supervision, Writing – review & editing, Methodology. PB: Methodology, Supervision, Writing – review & editing. EV: Supervision, Writing – review & editing. JB: Conceptualization, Funding acquisition, Methodology, Project administration, Supervision, Writing – original draft.
